# Type 2 Diabetes Biomarkers of Human Gut Microbiota Selected via Iterative Sure Independent Screening Method

**DOI:** 10.1371/journal.pone.0140827

**Published:** 2015-10-19

**Authors:** Lihua Cai, Honglong Wu, Dongfang Li, Ke Zhou, Fuhao Zou

**Affiliations:** 1 Wuhan National Laboratory for Optoelectronics, Huazhong University of Science and Technology, Wuhan, Hubei, China; 2 School of Information Science, Guangdong Ocean University, Zhanjiang, Guangdong, China; 3 Binhia Genomics Institute, BGI-Tianjin, BGI-Shenzhen, Tianjin, China; Wayne State University, UNITED STATES

## Abstract

Type 2 diabetes, which is a complex metabolic disease influenced by genetic and environment, has become a worldwide problem. Previous published results focused on genetic components through genome-wide association studies that just interpret this disease to some extent. Recently, two research groups published metagenome-wide association studies (MGWAS) result that found meta-biomarkers related with type 2 diabetes. However, One key problem of analyzing genomic data is that how to deal with the ultra-high dimensionality of features. From a statistical viewpoint it is challenging to filter true factors in high dimensional data. Various methods and techniques have been proposed on this issue, which can only achieve limited prediction performance and poor interpretability. New statistical procedure with higher performance and clear interpretability is appealing in analyzing high dimensional data. To address this problem, we apply an excellent statistical variable selection procedure called iterative sure independence screening to gene profiles that obtained from metagenome sequencing, and 48/24 meta-markers were selected in Chinese/European cohorts as predictors with 0.97/0.99 accuracy in AUC (area under the curve), which showed a better performance than other model selection methods, respectively. These results demonstrate the power and utility of data mining technologies within the large-scale and ultra-high dimensional genomic-related dataset for diagnostic and predictive markers identifying.

## Introduction

The biomedical study indicates that the onset of type 2 diabetes is associated with human gut microbiota [1, 2]. However, the human gut microbiota is a complex ecosystem, including thousands of bacterial species. Such diversity and complexity brings out a big challenge for researchers to filter the particular categories out of huge candidates when only a few samples can be obtained. Identifying a small number of biomarkers of gut microbiota related to type 2 diabetes is significant. Law of parsimony in statistical viewpoint is meaningful here. Investigating only limited genes makes it possible for researchers to further discover their role in health and disease, and then give proper suggestion for precaution or treatment. How best to select the genes in such conditions puts forward a classical statistical problem which is variable selection in high dimensional feature space.

Modeling on high dimensional data with low sample size is becoming a growing characteristic in many areas of contemporary statistics, especially in genomics fields. There are two top concerned aspects about such problems, one is accuracy of estimated model on selected subset, and another is interpretability of selected signature predictors.

In recent years, a lot of contributions have been made and related works have been published on this issue. As pointed out by Fan [3], the main theoretical questions, including what the limits of the dimensionality that such methods can handle and how to evaluate the optimality of variable selection procedures, have still unresolved.

Regularization methods have been widely studied in high dimensional variable selection. Tibshirani proposed well-known lasso and fused lasso [4, 5]. Lasso introduces an L1-norm penalty to generate a sparse model. Sparsity demand comes frequently with high dimensional data, in which only a small part of features have correlation with the response, it means that quite a few features will have regression coefficient 0 in mathematics. Some other estimation procedures have been published to account for certain structures of variables [6, 7]. The elastic net, combines L1-norm and L2-norm penalties of lasso and ridge regression linearly, and is supposed to overcome the limitations of lasso [8]. Motivated by using prior biological knowledge in approaches, several graph guided regularization procedures have been proposed for model fitting and feature selection [9–11]. SCAD [Fan and Li (2001)], smoothly clipped absolute deviation, which can lead to a sparse model like lasso and it performs favorably when compared with lasso soft thresholding rule and hard-thresholding rule. For variable selection methods are known to be susceptible to perturbation of the training data, Meinshausen and Bühlmann proposed to select variables according to their probabilities to stabilize the model [12]. Based on resampling training data many times, only those variables with highly selected probabilities crossing all subsamples would be included in the model. Their method is supposed to produce a more stable selection in high dimensional space.

A major limitation of aforementioned methods is that these methods cannot perform well consistently in handling correlations between variables. The real difficulty of high dimensional model selection comes from the relations among the variables, which means some unimportant variables highly correlated with important variables would have higher correlation with response than those true variables that are weakly correlated with response. This will make us select a wrong model, and lead to completely wrong scientific conclusions. In this article, we introduce a helpful model selection procedure called iterative sure independence screening (ISIS) which is proposed by Fan might contribute to deal with the spurious relation problem partly [13]. We apply this method on type 2 diabetes data and execute an empirical comparison with other variable selection approaches in terms of estimation accuracy. The experimental research indicates that ISIS outperforms other modern methods on estimation performance. By inspecting the selected signature we may discover the biological process involved in health and disease.

## Materials and Methods

### Data

We have 2 gut microbiota datasets of type 2 diabetes, which are obtained from recently published results [1, 2]. One dataset is collected from 344 Chinese individuals (170 cases and 174 controls) and another is acquired from 145 European individuals (102 cases and 43 controls). More detailed information of datasets was listed in Table 1. For each individual 9,879,896 predicted genes have been marked and measured at the initial stage. Then we performed a quality control procedure on data. According to the biological function, features with lower abundance were discarded, those that whose relative abundance bigger than 1 in more than 90% of samples were retained, finally, 17,473 genes were processed for the following analysis. It should be pointed out that Chinese dataset is collected from males and females between 14 and 86 years old but European dataset is merely from females close to 70 years old. Previous work has revealed the big difference of gut microbiota between people who have different dietary structure [14]. Considering the discrepancy of datasets, we implement variable selection methods on Chinese and European datasets respectively.

**Table 1:** Data.

	Gender				Condition	
Race	Male	Female	Age(mean/sd)	BMI(mean/sd)	T2D	Normal
Chinese	190	154	47.58/14.51	27.11/4.55	170	174
European	0	145	70.41/0.71	23.35/3.48	102	43

Chinese and European gut microbiota datasets of type 2 diabetes (T2D) used in our work. The ‘sd’ means standard deviation. BMI means body mass index.

### Variable Selection methods in high dimensional data

Variable selection in high dimensional data has been discussed in many literatures. The motivation of applying iterative sure independence screening method in our work lies in the fact that genes highly related with others may not lead to the same medical condition. We should discover true relations between genes and diseases and filter out spurious relations. For empirical evaluation, we compare ISIS method with other two representative feature selection methods: mRMR and ensemble feature selection. These methods are applied on Chinese and European datasets respectively and return a set of genes. These genes then can be used to estimate a classifier to predict the class of any sample. We can also carry out biological analysis on selected signature.

### Minimum redundancy and maximum relevance feature selection

By using this method, some researchers have selected 50 biomarkers of gut microbiota related to type 2 diabetes on 145 Chinese individuals. They published their work in [2]. Here we reuse this method for comparison. The mRMR, short for minimal-redundancy-maximal-relevance, is a kind of mutual-information-based feature selection method and refers to select features in terms of two criterions’ combination (maximal relevance and minimal redundancy) [15, 16]. Most used methods select top ranking features based on mutual information without considering relationships among features. This could result in selected genes correlated and covering narrow regions in space. As maximal statistical dependency criterion is hard to implement, mRMR criterion is a feasible alternative. For categorical variables, mutual information is a useful measure of the level of “similarity” between genes. Given two random variables *x* and *y*, their mutual information is defined according to their probabilistic density functions *p*(*x*), *p*(*y*) and *p*(*x*,*y*):

$$I\left(x,y\right)\;=\;{\int^{\text{​}}}^{\text{​}}\int^{\text{​}}\;p\left(x,y\right)\text{ log}\frac{p\left(x,y\right)}{p\left(x\right)p\left(y\right)}dxdy$$

The maximal-relevance constraint means to search the best features which could “optimally” characterize the class label c. In our condition the class label c is the disease status (−1 or +1). Thus the maximum-relevance condition is to maximize the total relevance of all genes in *S*: $\max V_{I},V_{I}=\frac{1}{\left|S\right|}\sum_{i\in S}I(c,i)$, where *I*(*c*,*i*) denotes the mutual information between the feature *i* and the class label *c*, and ∣*S*∣ is the number of features in *S*. Intuitively, the feature are selected according to their discriminant power would likely have rich redundancy; they could hardly maximally represent the original space covered by the entire dataset. The minimal redundancy criterion is a supplement to maximal relevance. This condition could be added to select more representative features: $\min W_{I}=\frac{1}{|S{|}^{2}}\sum_{i,j\in S}I\left(i,j\right)$. The mRMR method selects features by optimizing both conditions equally. The combination of two conditions has two forms: (*V_I_* − *W_I_*) and max(*V_I_*/*W_I_*). We adopt the difference form to execute our experiment.

### Ensemble feature selection

The penalized logistic regression method is attractive in categorical applications involving high-dimensional data. The most commonly induced penalty is *L*_1_-norm (lasso) or the combination of *L*_1_-norm and *L*_2_-norm (elastic net). The lasso estimate *β* ∈ *R* ^*p*^ could be obtained by minimizing the objective function *L*(*β*) + *λ*∣∣*β*∣∣_1_, where L(*β*) is the empirical logistic loss, ${\left|\left|\beta \right|\right|}_{1}=\sum_{i=1}^{p}\left|\beta_{j}\right|$ is *L*_1_-norm penalty and λ is the tuning parameter which controls the sparsity of the estimate. Alternatively, the elastic net is similar to lasso but adds an *L*_2_-norm penalty to objective function $L(\beta )+\lambda \alpha ||\beta |{|}_{1}+\frac{1}{2}\lambda (1-\alpha )||\beta |{|}_{2}^{2}$, where $||\beta |{|}_{2}^{2}=\sum_{i=1}^{p}\beta_{j}^{2}$ is *L*_2_-norm penalty and *λ*, *α* are two tuning parameters. In both methods, the parameter *λ* determines the sparsity of the estimated model in a same way. The model will become sparser when the parameter *λ* increases. The elastic net is supposed to be more stable in handling the correlated features.

In variable selection procedure, the optimal value of tuning parameters is often explored by CV (cross validation). As mentioned in many literatures, for high-dimensional data CV is prone to select too many variables including noise variables. Furthermore, embedded variable selection methods in high feature space, i.e., lasso and elastic net, are known to be sensitive to small perturbations of training data. In order to get a robust subset for prediction and generalization, we apply ensemble idea on embedded variable selection procedures. For lasso and elastic net, we add an additional aggregation strategy for stable feature selection. We first bootstrapped the samples 100 times (i.e. draw a subsample of size [n/2] from the data with replacement *M* times) and apply embedded selection method on each subsample to get *M* rankings (*R* ^1^,*R* ^2^,…,*R* ^*M*^) of all features. For lasso and elastic net, the ranking is the order in which the variables enter the selected subset when tuning parameter *λ* decreases. We then aggregated the *M* lists by computing a score $score\left(j\right)=\frac{1}{M}\sum_{m=1}^{M}R_{j}^{m}$ for each gene *j* as an average value of the function of its rank in each list. Here we employed stability selection criterion for aggregation and measured the percentage of bootstrap samples for which the gene ranks in the top *v*, i.e., *f*(*R*) = 1 if *R* ≤ *v*, 0 otherwise. For the implementation of penalized logistic regression method, we used the code implemented in the SLEP toolbox published along with Liu [17].

### Iterative sure independence screening approach

SCAD, smoothly clipped absolute deviation, means variable selection via noncave penalized likelihood which leads to a sparse model like lasso but it performs favorably [18]. Considering the linear regression model: *y* = *Xβ* + *ɛ*, where *y* = (*Y*_1_,*Y*_2_,…,*Y*_*n*_)^*T*^ is an *n*-vector of responses, *X* is an *n* × *p* matrix, *β* is a *p*-vector of estimators and *ϵ* is an *n*-vector of random noise. Fan and Li proposed SCAD penalty, which has first-order derivative

$${p_{\lambda }}^{\prime }\left(\beta \right)=\lambda \left\{I\left(\beta \leq \lambda \right)+\frac{{\left(a\lambda -\beta \right)}_{+}}{(a-1)\lambda }I\left(\beta >\lambda \right)\right\},$$

where *β* > 0, *a* > 2 and *I*(⋅) is an indicator function. Through a Bayesian risk analysis they recommended the best value for *a* is 3.7.

Sure independence screening (SIS), put forward by Fan [13], is based on correlation learning and aims that all important variables would survive the screening procedure with probability tending to 1. This method uses component-wise regression to identify important factors according to their marginal correlation with the responses. For aforementioned linear regression model, the vector of marginal correlations of predictors with responses can be computed by formula $\varpi =X^{T}y$. Then the *p* absolute components of ϖ are sorted in a descending order and a cutoff value *s* ∈ (0,1) is to be set to define a submodel,

$$M^{s}=\left\{1\leq i\leq p:\left|\varpi_{i}\right|is\text{ among the first }\left[sn\right]\text{ largest of all}\right\},$$

where [sn] is the integer part of *sn*.

SIS procedure is suggested to be applied in the preliminary step before other variable selection method should be implemented. After performing SIS to decrease the dimension from ultra-high to a moderate low size, lower dimensional variable selection method, such as SCAD, can be used to complete the model selection. The combined techniques are called SIS-SCAD for simplicity.

SIS is well understood in orthogonal design matrices. But, in practical applications there are usually general design matrices where SIS is malfunctioning. To overcome weak points of SIS someway, Fan proposed the following iterative process to enhance the methodological power. The implementation process of ISIS-SCAD is as follows. First, a subset of *d*_1_ variables *S*_1_ = {*X*_*i*_1__,…,*X*_*i*_*d*_1___} is selected by SIS-SCAD. Then an n-vector of residuals is obtained by regressing the response *y* over *S*_1_. In the following step, the same process SIS-SCAD is applied to the *p* − *d*_1_ variables using those residuals as the responses, which results in a subset of *d*_2_ variables $S_{2}=\left\{X_{i_{{}_{1}}},...,X_{i_{d_{2}}}\right\}$. Keep on doing above steps until *k* disjoint subsets *S*_1_,…,*S*_*k*_ whose union $S={⋃}_{i=1}^{k}S_{k}$ has a size *d* are obtained.

To expand the scope of the applicability of sure independence screening methodology beyond the linear model, Fan and Samworth extended it to a general pseudo-likelihood framework, including logistic regression [19]. In this framework, the features are ranked according to the marginal likelihood instead of the correlation with the response. Consider the generalized loss function form,

$$Q\left(\beta_{0},\beta \right)=n^{-1}\sum_{i=1}^{n}L(Y_{i},\beta_{0}+x_{i}^{T}\beta ),$$

where *β* = (*β*_1_,…,*β*_*p*_) is the parameter vector and *β*_0_ is the intercept term, and $\left(x_{i}^{T},Y_{i}\right)$ are the observation and the response for the *i* ^*th*^ sample. *L* is the loss by using $\beta_{0}+x_{i}^{T}\beta$ to predict *Y*_*i*_. The marginal utility of feature j is defined by minimizing the following loss function,

$$L_{j}=\min_{\beta_{0},\beta_{j}}n^{-1}\sum_{i=1}^{n}L(Y_{i},\beta_{0}+X_{i}j\beta_{j}).$$

Only two parameters, *β*_0_ and *β*_*j*_, are need to fit this model. Thus the marginal utilities vector *L* = (*L*_1_,…,*L*_*p*_) can be computed quickly. The idea of SIS in this framework is selecting features by ranking the components of *L* in ascending order. The feature *j* will be selected if *L*_*j*_ is in the *k* smallest elements of *L*. Such process is commonly employed before other refined variable selection method would be applied, such as SCAD. The two-stage procedures are also called SIS-SCAD.

Likewise, iterative procedure is also effective in this framework. The manner of ISIS in general pseudo-likelihood framework is analogous to the least squares ISIS procedure. At the first step, a subset *M*_1_ of *k*_1_ features is extracted by applying two-stage SIS-SCAD procedures. The second round screening is implemented as follows, firstly the marginal utilities of other *p* − *k*_1_ features are computed with the following formula,

$$L_{j}^{\left(2\right)}=\min_{\beta_{0},\beta_{M_{1}},\beta_{j}}n^{-1}\sum_{i=1}^{n}L(Y_{i},\beta_{0}+x_{i,M_{1}}^{T}\beta_{M_{1}}+X_{ij}\beta_{j}),$$

where $j\in \bar{M_{1}}=\left\{1,...,p\right\}-M_{1}$ and *x*_*i*,*M*_1__ is the sub-vector of *x*_*i*_ consisting of those elements in *M*_1_, then the *p* − *k*_1_ features are sorted by ranking the elements of *L* ^(2)^ and a subset $M_{2}^{*}$ corresponding to the $k_{2}^{*}$ smallest elements is extracted, at last a subset *M*_2_ with *k*_2_ features is selected in this round by applying SCAD on the reduced data limited to $M_{1}\cup M_{2}^{*}$ features. The process can be repeated until a subset *M*_*l*_ with a pre-specified size *l* obtained. The algorithm was implemented in the R package SIS published along with Fan [13].

### Accuracy of selected genes

The prediction performance of selected genes could be measured by estimation accuracy of the classifier trained on data limited to selected genes. Without loss of generality, we test several popular classification algorithms. More precisely, we employ four algorithms: logistic regression (LR), linear discriminant analysis (LDA), Naïve Bayes (NB) and support vector machine (SVM). In statistics, ROC (receiver operating characteristic) curve, is a graphical plot that illustrates the performance of a binary classifier. We employ the area under the ROC curve (AUC) to evaluate the discriminative power of classifiers. On both datasets, we perform a 10-fold CV (cross validation) procedure, where the classifiers are trained on 90% of the observations and the AUC is computed on the remaining 10%. In addition, to explore the influence of the size of selected genes on prediction accuracy, we compare the AUC of these classification algorithms executed on data restricted to different sizes of genes.

### Biological functional interpretability

To assess the biological function of these selected meta-markers, we annotated these markers and categorized them into GO term and KEGG pathway in order to detailedly describe the function of markers in the development of type 2 Diabetes. We downloaded the annotation dataset from website (http://meta.genomics.cn/metagene/meta/dataTools) [20], and then assigned the markers into the dataset with a perl script in-house and calculated the number of markers enriched in GO term and KEGG pathway.

## Results

To investigate the performance of ISIS-SCAD, we conducted an empirical comparison of this method with others in terms of estimation accuracy. We also executed biological function analysis on selected signature.

### Estimation accuracy

We implemented four variable selection methods on Chinese and European datasets respectively. In particular, they are mRMR, ensemble of lasso, ensemble of elastic net, and ISIS-SCAD. The first problem that needs to be solved is how many genes we should select from the candidates since the optimal subsets obtained by different variable selection methods may vary in amount. It is obvious that we cannot check the prediction performance trained on all sizes of selected genes. Technically we just tested the discriminative power of top *t* genes obtained by each variable selection method. For mRMR and ensemble feature selection, we can get a subset of genes with appointed size directly. As usual, we assigned the initial value 10 to *t*, and then we incremented *t* by 10 until it ended up with 100.

For ISIS-SCAD, the amount of selected genes is random with different parameter settings due to the implementation of this method itself. Thus we cannot obtain a subset of candidates with a specified size like mRMR and ensemble feature selection. By testing a few parameters, we discovered that the returned size of signature is random and fluctuates irregularly and it does not increase much no matter how the parameter varies. Due to its high time complexity it is impossible for us to test the whole parameter space, we just checked some typical parameters. After testing lots of parameters, the maximum size of selected genes we have got for Chinese data is 63, for European data the maximum size is 36. It is technically feasible for us to fix the numbers in a monotonic sequence to assess the influence of the numbers of genes used to estimate the model. For Chinese dataset, the size sequence is {10, 15, 18, 23, 26, 28, 34, 41, 43, 48, 50, 61, 63}. For European dataset, the size sequence is {4, 11, 15, 22, 24, 26, 27, 28, 29, 32, 34, 35, 36}.

In Table 2, we reported the accuracy (in AUC) of top *t* genes over Chinese dataset from combinations of four classification algorithm with mRMR and ensemble methods in a 10-fold cross-validation setting. In Table 3 we report the accuracy (in AUC) of selected signatures over Chinese dataset obtained from ISIS-SCAD with four classification algorithm in a 10-fold cross-validation setting. In the same way, we reported the accuracy obtained over European dataset in Tables 4 and 5.

**Table 2:** AUC obtained by mRMR and ensemble methods (Chinese).

Classifier	Method	10	20	30	40	50	60	70	80	90	100
SVM	mRMR	0.75	0.73	0.74	0.76	**0.77**	0.72	0.72	0.73	0.73	0.71
	Ensemble (lasso)	0.79	0.84	0.85	0.89	0.90	0.89	**0.91**	0.91	0.88	0.90
	Ensemble (Enet)	0.79	0.85	0.86	0.90	**0.91**	0.91	0.88	0.89	0.90	0.90
LR	mRMR	0.75	0.75	**0.78**	0.75	0.75	0.75	0.72	0.70	0.71	0.72
	Ensemble (lasso)	0.80	0.83	0.86	0.88	**0.89**	0.88	0.85	0.82	0.77	0.79
	Ensemble (Enet)	0.79	0.85	0.86	0.90	**0.91**	0.89	0.86	0.79	0.79	0.82
LDA	mRMR	0.75	0.75	**0.76**	0.72	0.74	0.71	0.68	0.69	0.70	0.69
	Ensemble (lasso)	0.80	0.84	0.87	0.90	0.89	0.90	0.91	**0.92**	0.92	0.92
	Ensemble (Enet)	0.80	0.86	0.87	0.90	0.91	0.91	0.90	0.91	**0.92**	0.91
NB	mRMR	0.72	0.71	0.74	0.72	0.75	0.76	0.75	0.76	0.76	**0.77**
	Ensemble (lasso)	0.76	0.85	0.87	0.88	0.88	0.89	0.89	**0.90**	0.88	0.89
	Ensemble (Enet)	0.78	0.85	0.86	0.87	**0.89**	0.88	0.88	0.89	0.89	0.89

AUC of *t*-gene (*t* is from 10 to 100) signatures obtained from each combination of classification algorithms and mRMR, ensemble of lasso, ensemble of elastic net, in a 10-fold cross validation over Chinese dataset. For each combination of classifiers with variable selection methods, we highlighted the best result of the minimum number of genes.

**Table 3:** AUC obtained by ISIS-SCAD (Chinese).

Classifier	10	15	18	23	26	28	34	41	43	48	50	61	63
SVM	0.71	0.80	0.83	0.82	0.89	0.93	0.92	0.91	0.96	**0.97**	0.95	0.96	0.96
LR	0.73	0.81	0.85	0.83	0.90	**0.93**	0.91	0.92	0.92	0.89	0.86	0.89	0.88
LDA	0.68	0.79	0.84	0.80	0.88	0.92	0.92	0.92	0.95	**0.96**	0.95	0.96	0.95
NB	0.69	0.71	0.77	0.79	0.78	0.83	0.82	0.87	0.82	0.85	0.85	**0.90**	0.88

AUC of signature size in {10, 15, 18, 23, 26, 28, 34, 41, 43, 48, 50, 61, 63}, combined with four classification algorithms in a 10-fold cross-validation. For each classification method, we highlighted the best result.

**Table 4:** AUC obtained by mRMR and ensemble methods (European).

Classifier	Method	10	20	30	40	50	60	70	80	90	100
SVM	mRMR	0.86	**0.89**	0.87	0.82	0.83	0.80	0.81	0.83	0.84	0.83
	Ensemble (lasso)	0.87	0.88	0.87	0.87	0.92	**0.94**	0.93	0.92	0.89	0.88
	Ensemble (Enet)	0.85	0.89	**0.91**	0.87	0.89	0.88	0.81	0.89	0.88	0.89
LR	mRMR	0.84	**0.88**	0.80	0.75	0.79	0.73	0.69	0.66	0.69	0.63
	Ensemble (lasso)	**0.86**	0.84	0.76	0.73	0.84	0.78	0.76	0.73	0.69	0.63
	Ensemble (Enet)	0.87	**0.89**	0.81	0.74	0.79	0.76	0.75	0.71	0.77	0.73
LDA	mRMR	0.80	0.84	**0.88**	0.80	0.78	0.76	0.75	0.69	0.67	0.64
	Ensemble (lasso)	**0.85**	0.85	0.83	0.79	0.77	0.74	0.74	0.74	0.71	0.67
	Ensemble (Enet)	0.86	0.88	**0.90**	0.79	0.76	0.79	0.75	0.76	0.77	0.69
NB	mRMR	0.79	0.76	0.78	**0.80**	0.78	0.77	0.76	0.77	0.77	0.76
	Ensemble (lasso)	0.85	0.87	**0.89**	0.87	0.84	0.83	0.85	0.81	0.77	0.75
	Ensemble (Enet)	0.88	0.89	0.88	0.88	**0.91**	0.89	0.89	0.88	0.87	0.90

AUC of *t*-gene (*t* is from 10 to 100) signatures obtained from each combination of classification algorithms and mRMR, ensemble of lasso, ensemble of elastic net, in a 10-fold cross validation over European dataset. For each combination of classifiers with variable selection methods, we highlighted the best result of the minimum number of genes.

**Table 5:** AUC obtained by ISIS-SCAD (European).

Classifier	4	11	15	22	24	26	27	28	29	32	34	35	36
SVM	0.77	0.91	0.87	0.97	**0.99**	0.97	0.98	0.97	0.94	0.96	0.94	0.92	0.94
LR	0.78	0.94	0.86	0.87	**0.98**	0.95	0.86	0.95	0.88	0.89	0.85	0.83	0.85
LDA	0.75	0.85	0.87	0.97	**0.99**	0.97	0.97	0.97	0.94	0.96	0.94	0.91	0.95
NB	0.74	0.81	0.86	0.90	0.92	0.83	0.89	0.83	**0.94**	0.92	0.93	0.88	0.90

AUC of signature size in {4, 11, 15, 22, 24, 26, 27, 28, 29, 32, 34, 35, 36}, combined with four classification algorithms in a 10-fold cross-validation. For each classification method, we highlighted the best result.

Globally, we observe in Tables 2 and 4 that ensemble feature selection method outperform mRMR over two different datasets significantly, for any given classification method. In addition, we perceive that there is no significant difference between ensemble of lasso and ensemble of elastic net over Chinese dataset but ensemble of lasso do a little bit well over European dataset. Thus we decide to choose ensemble methods for latter discussions with ISIS-SCAD. Second, we also observe that, among all classification algorithms, the SVM classifier consistently present good results when compared to other classifiers on both datasets. Therefore we choose SVM as a default classifier for further assessment of the performance of the selected signatures. Moreover, by comparing details from Table 2 to Table 5, we found that ISIS-SCAD outperforms other variable selection methods no matter which classifier we choose.

**Fig 1:**
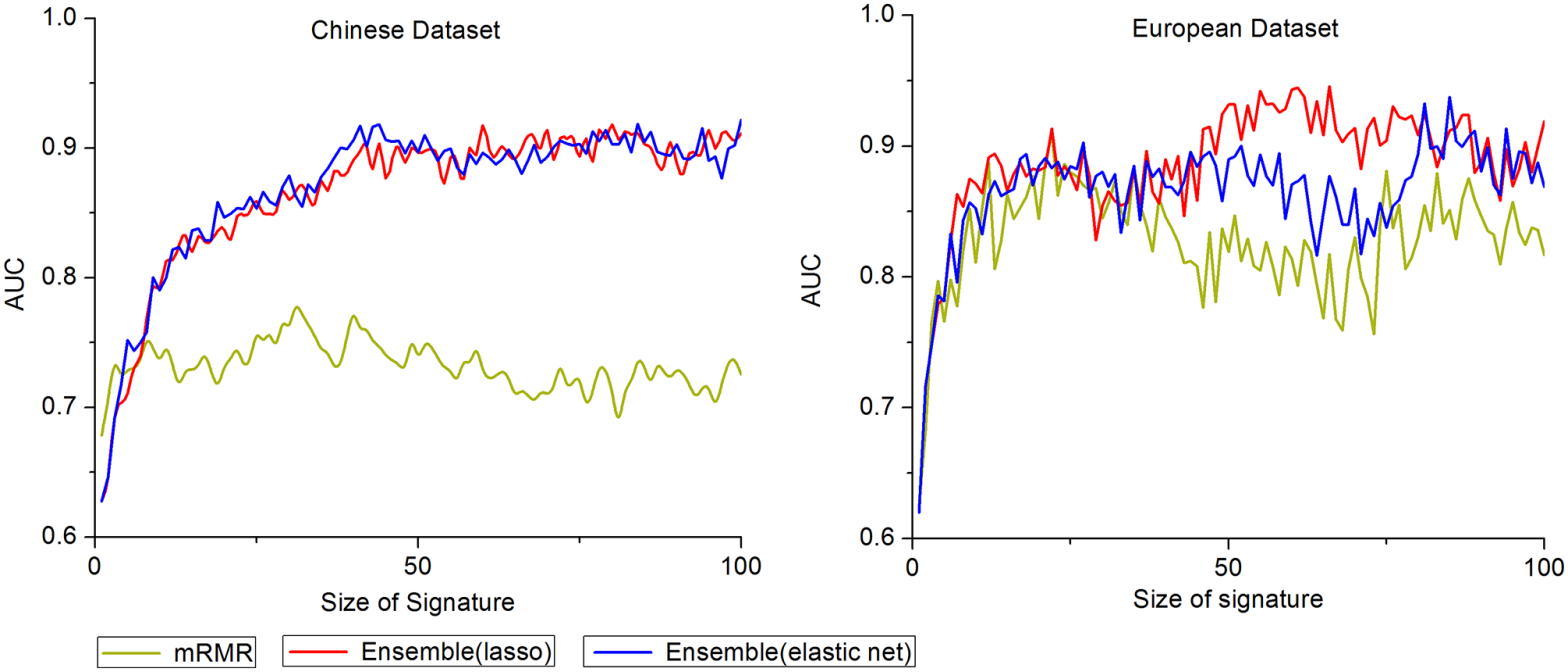
AUC. SVM classifier trained as a function of the size of signature, for mRMR, ensemble of lasso and ensemble of elastic net, in a 10-fold cross-validation setting on Chinese and European datasets respectively.

**Fig 2:**
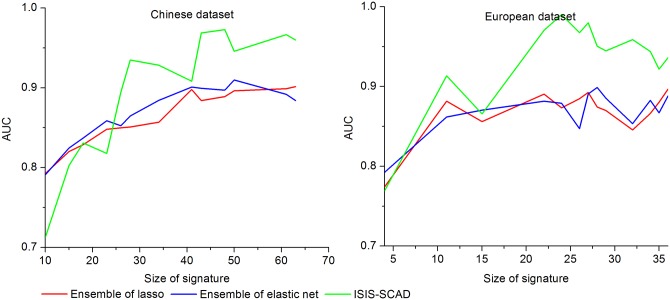
AUC obtained from SVM classifier estimated on genes selected by ISIS-SCAD and ensemble feature selection. Signature of size in {10, 15, 18, 23, 26, 28, 34, 41, 43, 48, 50, 61, 63} on Chinese dataset and size in {4, 11, 15, 22, 24, 26, 27, 28, 29, 32, 34, 35, 36} on European dataset in a 10-fold cross-validation setting.

In order to assess the influence of the size of signature on the estimation performance intuitively, we plot in Fig 1 the AUC of mRMR and ensemble feature selection methods, combined with a SVM classifier, as a function of the size of the signature. Likewise, Fig 2 depicts graphically the AUC reached by SVM classifier in Tables 3 and 5 separately. In order to perform a persuasive comparison, the AUC computed by SVM classifier estimated on size-fixed signatures obtained by ensemble feature selection methods in the same setting is also shown in Fig 2.

As regards the influence of the size of selected signatures, for any given variable selection method, we notice that for both datasets the AUC rises early in Figs 1 and 2, implying that fewer than 100 genes may be sufficient to obtain the maximal performance. Moreover, it is worth noticing that the AUC of any method is not utterly monotonic and fluctuates to a small extent when it reaches a certain height. For mRMR method, increasing the number of features may lead to a decreasing AUC apparently. Undoubtedly, to avoid introducing too many redundant features it is best for us not to choose 100 as the signature size.

The optimal AUC we have got on Chinese dataset is 0.97 with 48-gene obtained by ISIS-SCAD. But the best result of ensemble feature selection method is 0.91 that is estimated on 60 genes. For European dataset, we got the peak AUC 0.99 with 24-gene reached by ISIS-SCAD. And the highest AUC reached by ensemble feature selection is 0.94 that is computed on 60 genes. Obviously, the ISIS-SCAD gives higher prediction performance with fewer marker genes than ensemble feature selection methods, especially on European dataset.

**Fig 3:**
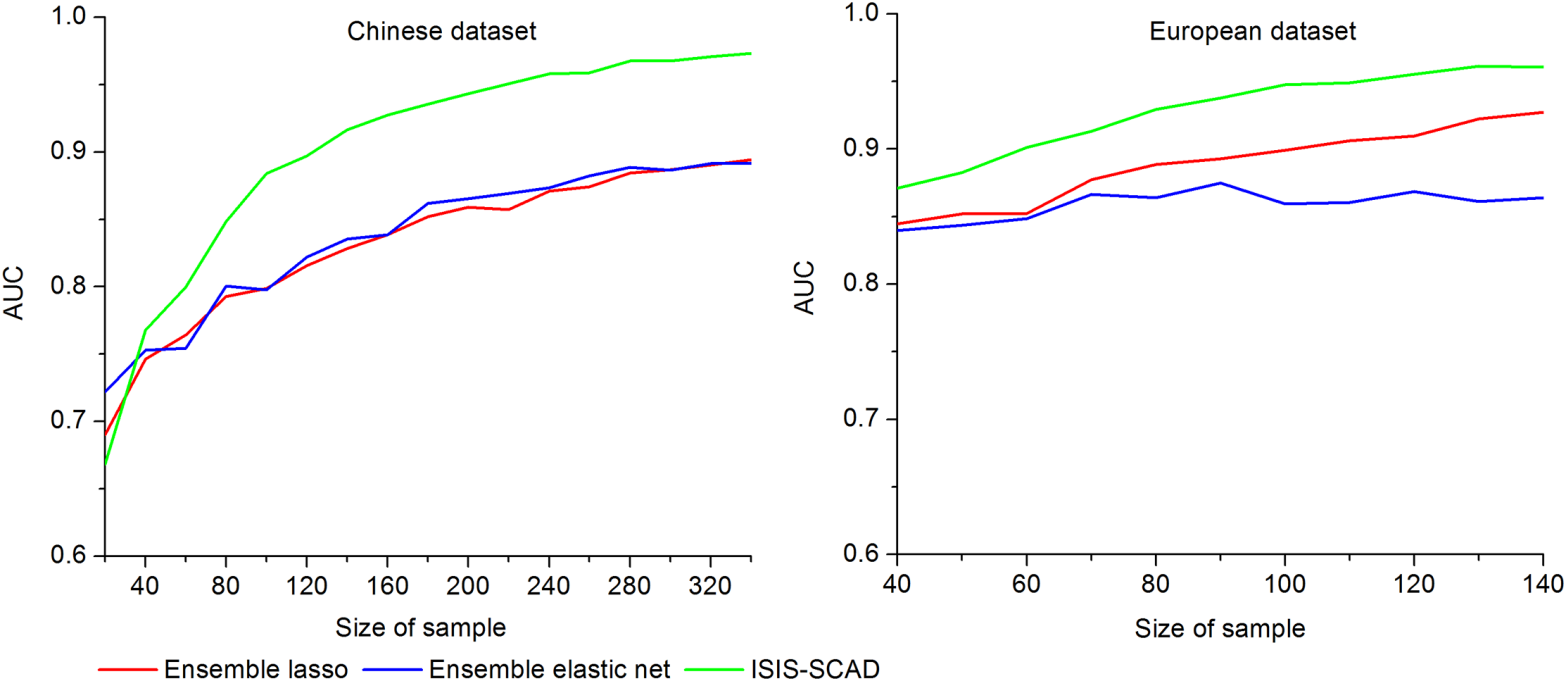
Averaged AUC obtained from SVM classifier combined with three variable selection methods. SVM classifier estimated as a function of sample size in a 50 × 10-fold cross-validation setting. We show accuracy of 60-gene of ensemble feature selection and 48-gene of ISIS-SCAD on Chinese dataset. For European dataset, the accuracy of ensemble feature selection is computed on 60-gene and the accuracy of ISIS-SCAD is on 24-gene.

In order to assess the stability and robustness of variable selection method, we also implement experiments to measure the influence of the sample size on estimating accuracy. For each variable selection, we repeated 50 times computing the 10-fold cross-validation AUC reached with a SVM classifier as a function of sample size. Fig 3 shows the average AUC obtained from three variable selection methods on each dataset separately.

Clearly, we observe that the average accuracy increases with the number of samples for each variable selection method on Chinese dataset. And the relative order of all methods is almost independent of the sample size in this dataset. The same thing happens in European dataset on ISIS-SCAD and ensemble of lasso. However, the performance of ensemble elastic net on European dataset seems to be insensitive to the number of samples. No matter how the influence of sample size would be, we always notice that ISIS-SCAD outperforms ensemble feature selection on both datasets consistently.

We also test how the selected signature behaves on another dataset. In a 10-fold cross-validation setting, the best AUC from Chinese dataset estimating on European signatures is 0.62 reached by the combination of ISIS-SCAD and logistic regression classifier. In the same way, the optimal AUC on European dataset is 0.61 achieved by the same combination. The experiment result shows the performance is very poor when the signature is selected on one dataset but estimated on another dataset. We also found there are no overlaps between selected signatures when they are from different datasets. But signatures obtained by different variable selection methods share a little overlap when they are produced in the same dataset. Considering the influence the gender, we extracted Chinese females individuals to form a subset and conducted variable selection methods on this subset. The result shows that there are still no overlaps between signatures obtained from Chinese and European females respectively. But the selected signatures obtained from Chinese female subset share 22% (mRMR), 21% (ensemble lasso), and 12.5% (ISIS-SCAD) genes with those signatures produced from the whole Chinese dataset. The reason why we neglected the influence of the age is because the number of Chinese females as the same age range of European females is too small.

### Biological interpretability

Functional analysis was performed mainly on KEGG Orthologue (KO) and eggNOG (evolutionary genealogy of genes: Non-supervised Orthologous Groups), both these two databases contain detailed information on biological pathway and functional categories.

Before the functional analysis, we annotated the sequence of 48 markers from Chinese dataset and 24 markers from Eurpean dataset to phylum level, and found 71% of markers belong to firmicutes, which was demonstrated that have a significant correlation with fat storage and energy harvest [21, 22]. we also found 67% of 24 markers belong to firmicutes in the European meta-markers, which demonstrates these markers we selected via our method have similar components (S1 Table).

Due to the limited number of markers, we cannot perform enrichment analysis of the pathway. But some KEGG pathway contains more marker-sequences, such as Genetic Information Processing, Cellular Processes and Signaling, Replicate and repair. More interesting, Glycan Biosynthesis and Metabolism was reported involved in T2D development and play a significant role in the pathobiology of obesity and T2D, were found containing two marker sequences [23].

On the whole, these two sets selected from different cohorts have tiny difference in biological functionality. To comprehensively evaluate the selected biomarkers in detail, we respectively ranked two gene sets in a descendant order according to the absolute coefficients of the estimated model. One marker of top 5 from Chinese that named MH0262_GL0011555 involved in the category of eggNOG plays an important role in translation, ribosomal structure and biogenesis. Another interesting marker from Chinese top 5 was located in cell cycle control, cell division, and chromosome partitioning. For European set, one marker that different from Chinese was involved in Signal transduction mechanisms, which was demonstrated involved in the development of diseases, such as Alzheimer [24].

## Simulations

Though ISIS-SCAD performs well on type 2 diabetes datasets, we are not sure that whether it could keep the performance under some certain scenarios. We conducted simulation studies to further examine the performance of ISIS-SCAD. We mainly focused on two issues. First, ISIS-based method aims to capture the linear relationship between the response variable and the predictor. Thus ranking marginal utilities probably would work weakly for nonlinear relationships. Second, an important variable is marginal weakly correlated with but jointly contributes to the response. We wondered that whether ISIS-SCAD still works well under these scenarios.

### Simulated Example I

To inspect the performance of ISIS-SCAD over nonlinear relationships to a limited extend, we used the model as follows,

$$y=5X_{1}+5X_{2}^{2}+5X_{3}^{3}+\varepsilon ,$$

where *X*_1_,…,*X*_*p*_ are *p* predictors and *ɛ* ∼ *N*(0,1) is noise and independent of *p* predictors. We simulated 100 data sets for every model. Each data set consisting of 50 observations is drawn from a multivariate normal distribution *N*(0,Σ), where Σ = (*σ*_*ij*_)_*p*×*p*_ is a covariance matrix that has entries *σ*_*ii*_ = 1,*i* = 1,…,*p*, and *σ*_*ij*_ = *ρ*,*i* ≠ *j*. We tested 6 models by different combinations of *p* = 100,*p* = 1000 and *ρ* = 0.1,*ρ* = 0.5,*ρ* = 0.9.

For each model, we employed ISIS-SCAD to select 20 variables and tested their accuracy of containing the true model {*X*_1_,*X*_2_,*X*_3_}. We reported the probabilities of ISIS-SCAD that includes the true model in Table 6.

**Table 6:** Results of simulated example I: accuracy of ISIS in including the true model {*X*_1_,*X*_2_,*X*_3_}.

	*ρ* = 0.1	*ρ* = 0.5	*ρ* = 0.9
*p* = 100	0.21	0.16	0.06
*p* = 1000	0.02	0.01	0

Accuracy of ISIS on different correlation *ρ* and dimensionality *p* setting under nonlinear relationship. For each model, 100 data sets consisting of 50 observations were simulated and 20 variables were selected for computing the accuracy.

On the whole, ISIS-SCAD performs poorly under our nonlinear setting here. However, we still observed that high dimensionality and high collinearity degenerate the performance of ISIS-SCAD. Certainly, due to the diversity of nonlinear relationships, there are no good measures to fully characterize the strength of the nonlinear relationships. It is clear that ISIS-SCAD cannot pick up the true model precisely under nonlinear conditions.

### Simulated example II

For the second issue, we used the same model as the example in [13] as follows,

$$y=5X_{1}+5X_{2}+5X_{3}-15\rho^{1/2}X_{4}+\varepsilon ,$$

where *X*_4_ ∼ *N*(0,1) and has correlation *ρ* ^1/2^ with rest *p* − 1 variables. The other set-up of this example is as same as the simulated example I. The variable *X*_4_ is marginally uncorrelated with but jointly contributes to the response *y*. We reported the percentage of ISIS-SCAD that includes the true model {*X*_1_,*X*_2_,*X*_3_,*X*_4_} in Table 7.

**Table 7:** Results of simulated example II: accuracy of ISIS in including the true model {*X*_1_,*X*_2_,*X*_3_,*X*_4_}.

	*ρ* = 0.1	*ρ* = 0.5	*ρ* = 0.9
*p* = 100	1	1	0.97
*p* = 1000	0.98	0.97	0.81

Accuracy of ISIS on different correlation *ρ* and dimensionality *p* setting under jointly contribution scenario. 100 data sets consisting of 50 observations were simulated and 20 variables were selected for computing the accuracy.

In this simulation, ISIS-SCAD can pick up true variables with high probabilities. It demonstrates that ISIS-SCAD can effectively handle the second issue. We also perceived the adverse influence of high collinearity and high dimensionality here.

## Discussion

In this article, we introduced a useful procedure called ISIS for variable selection in high dimensional gene expression data. We implemented this method on Chinese and European T2D data respectively and compared it with several variable selection methods on estimation accuracy. And we analyzed biological functionality of selected signature.

The bottleneck problem of variable selection in high dimensional data lies in the correlation between features. Common variable selection methods tend to select more spurious features. But ISIS-SCAD can partially deal with the relations between variables in high dimensional space. We have noticed that in both Chinese and European datasets, ISIS-SCAD outperforms ensemble feature selection in terms of accuracy remarkably. And ISIS-SCAD achieves the better performance with a smaller subset than ensemble feature selection.

As regards the choice of the sizes in sequence for ISIS-SCAD, we need to explain that it is impossible for us to search the whole parameter space, thus we can only choose the numbers among those we have got by ISIS-SCAD. The important thing we considered is to discover the influence of the size of signature on prediction performance. We tested the prediction accuracy of all selected subsets and found that the prediction accuracy rises roughly when the size of signature increases. When the size of signature is greater than 40 the AUC estimated on such subset is no less than 0.9. In fact, in the process of testing different parameters, we have found that similar parameters yield similar or even same subsets. This means that the subsets would share most or all genes when they are produced from successive parameters. Another thing should be mentioned is that some features are selected with probability tending to 1. We supposed that we need not list all numbers in our sequence but pick up those representative ones. Our principle for choosing the numbers in the sequence is to keep the consistency of the selected subsets as much as possible. We would choose the subset which shares more genes with the precursor and the successor in the monotonic size sequence. Take Chinese dataset as example, we started from the minimum size 10, ended up at maximum size 63. We only got one subset with size 10. The rest numbers are chosen by following above-mentioned principle. Actually, even if we replace some numbers in the sequence, the tendency of the curve in Fig 2 will not change.

As regards the choice of classifier used to estimating selected features, we found that the SVM classifier give the best and stable accuracy on both datasets. By carefully checking the details in Tables 2 and 3, we perceived that none of the accuracy is always increasing in this range of signature sizes for each combination of variable selection methods and classification algorithms. Especially, given any variable selection method, we noticed that on European dataset the AUC computed by LR, LDA and NB decreases markedly when the size of signature increases, and the AUC obtained from SVM trends to decrease to a little lighter extent. Similarly, for each variable selection method, the AUC on Chinese dataset by LR decreases clearly. Though the AUCs on Chinese dataset by other three classification algorithms do not trend to decrease remarkably but all of them reach the peak with features less than 100. This fact suggests that there may be some overfitting and the number of true features is likely less than 100. From details in our experimental results, we presume that few than 63 genes is enough to present powerful discriminative capabilities for Chinese dataset and top 36 genes lead to significantly good performances in general for European dataset. It confirms the well-formedness of our parameter settings of ISIS-SCAD.

Furthermore, it is important for us to observe that the selected signatures from Chinese and European share no overlap, and the selected signature performs poor when it is extracted from one dataset but tested on another. Though the selected signatures have no overlap when they are from different races, they may encode the same biological functionality. This suggests that it is helpful to extract information involved in the biological processes from the gene list. This result also demonstrates the difference of gut microbiota between Chinese and European people on the other side.

In according with the advance of algorithm and sequencing technology, more interesting will be discovered based on the existing datasets. This article provides a cue to mining the biological datasets with algorithms from computational industry.

## Supporting Information

S1 Table Biological analysis of markers selected from Chinese and European data. 48 makers from Chinese and 24 markers from European were annotated and analyzed on KEGG pathway and eggNOG function.
(XLS)
